# *Vibrio parahaemolyticus* Foodborne Illness Associated with Oysters, Australia, 2021–2022

**DOI:** 10.3201/eid3011.240172

**Published:** 2024-11

**Authors:** Emily Fearnley, Lex E.X. Leong, Alessia Centofanti, Paul Dowsett, Barry G. Combs, Anthony D.K. Draper, Helen Hocking, Ben Howden, Kristy Horan, Mathilda Wilmot, Avram Levy, Louise A. Cooley, Karina J. Kennedy, Qinning Wang, Alicia Arnott, Rikki M.A. Graham, Vitali Sinchenko, Amy V. Jennison, Stacey Kane, Rose Wright

**Affiliations:** South Australian Department for Health and Wellbeing, Adelaide, South Australia, Australia (E. Fearnley, A. Centofanti); University of South Australia, Adelaide (L.E.X. Leong); SA Pathology, Adelaide (L.E.X. Leong, H. Hocking); Department of Primary Industries and Regions, Adelaide (P. Dowsett); Department of Health Western Australia, Perth, Western Australia, Australia (B.G. Combs); Northern Territory Centre for Disease Control, Darwin, Northern Territory, Australia (A.D.K. Draper); The Peter Doherty Institute for Infection and Immunity, Melbourne, Victoria, Australia (B. Howden, K. Horan, M. Wilmont); PathWest Laboratory Medicine, Perth (A. Levy); Royal Hobart Hospital, Hobart, Tasmania, Australia (L.A. Cooley); Canberra Hospital and Health Services, Canberra, Australian Capital Territory, Australia (K.J. Kennedy); Westmead Hospital, Westmead, New South Wales, Australia (Q. Wang); The University of Sydney, Sydney, New South Wales, Australia (A. Arnott, V. Sintchenko); Queensland Health Forensic and Scientific Services, Coopers Plains, Queensland, Australia (R.M.A. Graham, A.V. Jennison); Australian Government Department of Health and Aged Care, Canberra (S. Kane, R. Wright)

**Keywords:** *Vibrio parahaemolyticus*, food poisoning, oysters, Australia, foodborne illness, bacteria, food safety, enteric infections

## Abstract

The bacterium *Vibrio parahaemolyticus* is ubiquitous in tropical and temperate waters throughout the world and causes infections in humans resulting from water exposure and from ingestion of contaminated raw or undercooked seafood, such as oysters. We describe a nationwide outbreak of enteric infections caused by *Vibrio parahaemolyticus* in Australia during September 2021–January 2022. A total of 268 persons were linked with the outbreak, 97% of whom reported consuming Australia-grown oysters. Cases were reported from all states and territories of Australia. The outbreak comprised 2 distinct strains of *V. parahaemolyticus,* sequence types 417 and 50. We traced oysters with *V. parahaemolyticus* proliferation back to a common growing region within the state of South Australia. The outbreak prompted a national recall of oysters and subsequent improvements in postharvest processing of the shellfish.

*Vibrio parahaemolyticus* is a marine and estuarine bacterium that is ubiquitous in tropical and temperate waters worldwide (*1*). Infections, including wound infections, can occur in humans through exposure to water, and enteric infections are attributed most commonly to ingestion of raw or undercooked seafood. Invasive bloodstream infections and death rarely occur (*2*,*3*). Human infection is not associated with person-to-person spread or transmission through the fecal–oral route, and environmental presence of *V. parahaemolyticus* has not been linked to fecal contamination (*4*). The virulence of *V. parahaemolyticus* is associated with the presence of a thermostable direct hemolysin (coded by *tdh* gene) or thermostable related hemolysin (*trh* gene) (*5*).

Foodborne *V. parahaemolyticus* outbreaks have been reported across Asia, the United States, South America, Europe, and New Zealand, predominantly associated with consumption of filter-feeding bivalve shellfish, such as oysters and mussels (*6*–*12*). *V. parahaemolyticus* infections show seasonal patterns, with increases in warmer months, because *V. parahaemolyticus* will grow in seawater at temperatures >14°C–19°C (*6*). Climate change can increase the distribution and incidence of *V. parahaemolyticus* (*6*,*13*), and a rise in water temperature is the main environmental factor associated with growth of *V. parahaemolyticus* in oysters. Postharvest risk reduction strategies focus on rapid cooling and maintaining cold refrigeration of oysters throughout the supply chain to prevent bacterial growth, which occurs at air temperatures >10°C (*14*).

Until recently, foodborne outbreaks of *V. parahaemolyticus* were rare in Australia. Only 4 outbreaks were reported during 2002–2019, affecting a total of 24 persons (*15*). Two previously reported outbreaks were linked to consumption of oysters; 1 from oysters produced in Tasmania and 1 from oysters grown in South Australia. Only 29 locally acquired, sporadic foodborne cases of *V. parahaemolyticus* were reported in Australia in 2016–2020; 22 of the infected persons reporting oyster consumption (76%) (*15*). *V. parahaemolyticus* infections might be underreported in Australia because pathology laboratories rarely include it in routine fecal testing procedures and the infection is a notifiable condition in only 4 of the 8 states and territories of Australia.

In September 2021, at the end of the winter season in the Southern Hemisphere, health officials identified an increase in locally acquired *V. parahaemolyticus* cases in South Australia, and a similar trend was later noted in other jurisdictions of Australia. In November 2021, through the OzFoodNet network, a multijurisdictional outbreak investigation commenced to coordinate the public health response. OzFoodNet is a network of epidemiologists across Australia who are responsible for undertaking surveillance and outbreak investigations of foodborne disease (*16*). Investigators worked closely with jurisdictional public health laboratories and with the Australia food regulatory authorities who implement control measures.

## Methods

*V. parahaemolyticus* infection is a notifiable disease under legislation in the Australia jurisdictions of the Northern Territory, South Australia, Tasmania, and Western Australia, where laboratories are required to report cases to their respective health departments. Public health authorities contacted diagnostic laboratories in the remaining Australia jurisdictions to request reported detections of *V. parahaemolyticus* in fecal specimens (under the auspices of an OzFoodNet multijurisdictional outbreak investigation).

### Epidemiologic Investigation

We defined an outbreak case as illness in any person with a fecal specimen testing positive for *V. parahaemolyticus* during September 7, 2021–February 18, 2022. We conducted a descriptive case series investigation, which entailed telephone interviews of case-patient using a standardized questionnaire to obtain demographic information (age, sex, jurisdiction of residence), onset of illness, symptoms, medications, risk factors, and consumption of seafood during the exposure period (defined as 7 days before onset).

We classified cases as confirmed outbreak cases if single-nucleotide polymorphism (SNP) cluster analysis was performed on sequence type (ST) 417 or ST50 *V. parahaemolyticus* isolates on AusTrakka (a national genomic surveillance platform) and determined to be highly related within each ST. We considered outbreak cases as probable if they were typed as ST417 or ST50 without further phylogenetic analysis on AusTrakka and as possible if isolates were unable to be further typed (no ST). Cases were excluded if case-patients had traveled overseas in the 7 days before onset, if another ST was identified, or if the sequences did not cluster by phylogenetic analysis on AusTrakka. We used a broad case definition to include both STs in the outbreak investigation to describe the overall increase in locally acquired *V. parahaemolyticus* cases.

We entered case data into REDCap v10.3.4 (Vanderbilt University, https://projectredcap.org). We calculated proportions, medians, and ranges by using Stata BE v17 (Stata, https://www.stata.com). Where data were missing, we calculated a proportion with known responses only.

### Environmental Investigations

Jurisdictional food regulatory authorities conducted traceback investigations for oyster exposures. Traceback activity revealed harvest area and dates, and investigators then sought information relative to temperature control. Food regulatory personnel collected oyster samples from case households and retail premises. The South Australian Shellfish Quality Assurance Program collected oyster samples direct from growers. Technicians processed and tested oyster samples in approved laboratories across jurisdictions to determine the presence of *V. parahaemolyticus* according to the Australian standards for food microbiology examination for specific organisms. Methods involved grinding 25 g of oysters with alkaline peptone water before overnight incubation at 36°C ± 2°C. Laboratory technicians isolated *V. parahaemolyticus* from the ground samples on thiosulfate–citrate–bile salts–sucrose agar at 36°C ± 2°C. They collected positive green-colonies and sent the samples to public health laboratories for whole-genome sequencing (WGS).

### Genomic Sequencing and Analysis of *V. parahaemolyticus*

Public health laboratories in each jurisdiction sequenced *V. parahaemolyticus* isolates from cases and food samples for species confirmation and ST determination through multilocus sequence typing (https://github.com/tseemann/mlst) (*17*). Technicians performed WGS by using Illumina platforms (MiSeq and NextSeq 500/550; Illumina, https://illumina.com) with paired-end reads. Public health laboratories shared the raw sequencing reads of most isolates to AusTrakka (*18*). The national analysis team performed quality filtering, virulence gene detection, and phylogenetic analyses by using Snippy version 4.6.0 (https://github.com/tseemann/snippy) for cluster identification. Laboratory researchers determined genomic clusters for respective STs by using single-linkage clustering based on the SNP distance threshold of 5 SNPs, 10 SNPs, and 20 SNPs. The laboratories also performed pangenome analysis by using Roary version 3.13.0 (https://sanger-pathogens.github.io/Roary) (*19*) to include other publicly available *V. parahaemolyticus* genomes from PubMLST (https://pubmlst.org) and visualized a phylogenetic tree by using ggtree (https://guangchuangyu.github.io/software/ggtree). Genome sequences were uploaded to the National Center for Biotechnology Information’s Sequence Read Archive under the BioProject Accession nos. PRJNA1129299, PRJNA783474, PRJNA856407, and PRJNA1131944.

## Results

### Epidemiologic Investigation

**Figure 1:**
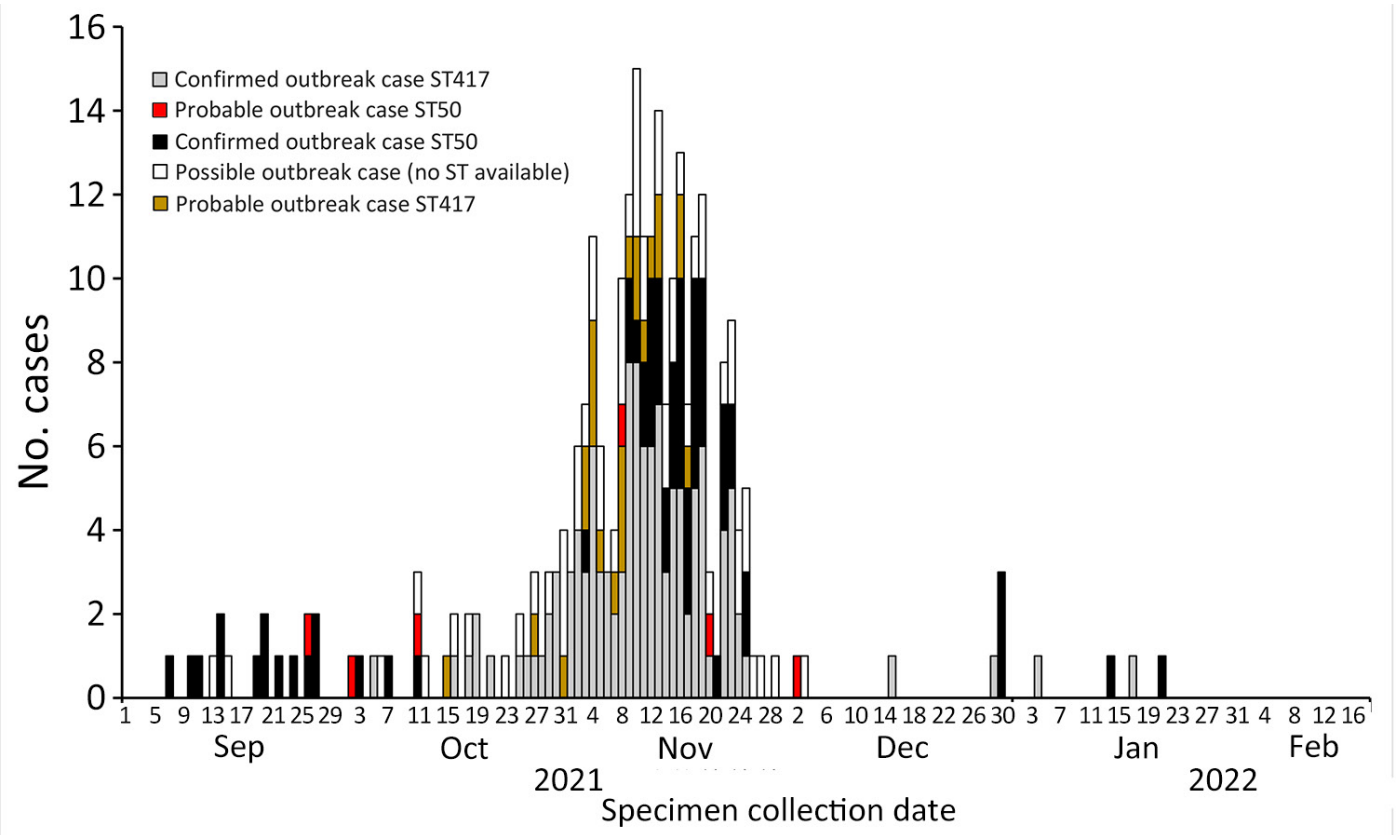
Epidemic curve of *Vibrio parahaemolyticus* outbreak cases by specimen collection date, outbreak case classification, and sequence typing, Australia, September 7, 2021–February 18, 2022. ST, sequence type.

We investigated a total of 268 outbreak cases from all Australia jurisdictions:184 confirmed cases, 29 probable cases, and 55 possible cases (Figure 1). The outbreak occurred over a 5-month period, and the peak of cases occurred in mid-November 2021. Infections of ST50 were reported initially, followed by predominant reports of ST417 infections; subsequent reports then revealed a period of high overlap of the 2 STs. We noted the highest percentage of reported cases from residents of South Australia (28%, n = 76), followed by 2 jurisdictions where *V. parahaemolyticus* is not notifiable, Victoria (26%, n = 69) and Queensland (22%, n = 59) (Table 1). Some case-patients reported spending their entire incubation period in other jurisdictions, including a Tasmania resident exposed in South Australia and a South Australia resident exposed in Western Australia. More case-patients were male (57%) than female (43%). All jurisdictions with >5 outbreak cases included cases of both ST50 and ST417. The median age of case-patients was 52 years (range 1–90 years) (Table 2). 

**Table 1:** *Vibrio parahaemolyticus* outbreak cases (by jurisdiction of residence and ST, Australia, September 7,2021–February 18, 2022*

Jurisdiction of residence	ST50	ST417	No ST available	Total no. (%) cases
South Australia	31	44	1	76 (28)
Victoria	23	46	0	69 (26)
Queensland	4	11	44	59 (22)
Western Australia	7	23	3	33 (12)
New South Wales	5	15	6	26 (10)
Australian Capital Territory	0	3	0	3 (1)
Tasmania	0	1	0	1 (0.4)
Northern Territory	0	0	1	1 (0.4)
Total	70	143	55	268

*ST, sequence type.

**Table 2:** *Vibrio parahaemolyticus* outbreak cases by demographic and clinical characteristics, Australia, September 7, 2021–February 18, 2022

Characteristic	No. (%) cases, n = 268
Sex	
M	152 (57)
F	115 (43)
Not stated	1 (0.4)
Age group, y	
0–9	1 (0.4)
10–19	3 (1)
20–29	12 (5)
30–39	38 (14)
40–49	64 (24)
50–59	58 (22)
60–69	59 (22)
70–79	23 (9)
>80	10 (4)
Symptoms*	
Diarrhea	206/206 (100)
Watery diarrhea	159/161 (99)
Abdominal pain	165/195 (85)
Lethargy	153/191 (80)
Nausea	138/200 (69)
Fever	98/203 (48)
Headache	96/198 (48)
Vomiting	71/204 (35)
Bloody diarrhea	8/181 (4)

*Values indicate number of case-patients who reported the symptom of those who answered the symptom question. Not all case-patients were asked about all symptoms.

Of those with available information, 25% of case patients (51/206) sought treatment at hospital emergency departments and 13% (27/209) of case-patients were hospitalized; emergency department information was not reported for 3 case-patients. Of 24 cases with length of hospitalization recorded, the median stay was 2.5 days (range 1–7 days). There were no deaths. Of those who responded to symptom-specific questions, all 195 case-patients interviewed reported diarrhea, and 85% (165) reported abdominal pain. One case-patient had *V. parahaemolyticus* isolated from both a fecal specimen and blood culture. The median duration of illness for 131 cases with data available was 7 days (range 1–17 days); however, 40 cases were still unwell at the time of interview and were therefore not included in this calculation.

Of 206 case-patients interviewed, 199 (97%) reported consuming oysters, 189 (92%) reported consuming at least some of the oysters raw, and 25 (12%) reported consuming oysters for >1 meal in the week before onset. For case-patients who consumed oysters on only a single occasion (n = 131), the median incubation period was 1 day (range 4 hours to 7 days). The median number of oysters eaten per case-patient was 6 (range 1–31 oysters). Case-patients purchased oysters at a range of venues, including restaurants (n = 71), supermarkets or seafood stores (n = 23), farms (n = 17), oyster tours (n = 9), and takeaway venues (n = 8). Exposure to other seafood was common, including 41% (n = 84) who reported consuming fish (varied types) and 37% (n = 76) who consumed prawns in the 7 days before onset. For those who consumed fish or prawns, most reported food to have been cooked. Less than 20% of case patients reported consuming seafood other than oysters, fish, or prawns (Table 3). From 166 case interviews, 25% (42) of case-patients reported they had taken medication that reduced stomach acid (e.g., reflux or ulcer medications) in the month before onset.

**Table 3:** *Vibrio parahaemolyticus* outbreak case-patients reporting exposure to seafood in the 7 days before onset of illness, Australia, September 7, 2021–February 18, 2022

Seafood	No. (%) cases exposed
Oysters	199 (97)
Oysters eaten raw	189 (95)
Fish*	84 (41)
Fish eaten raw	14 (17)
Prawns	76 (37)
Prawns eaten raw	4 (11)
Squid	37 (18)
Scallops	29 (14)
Mussels	19 (9)
Lobster/crayfish	17 (8)
Crab	13 (6)
Octopus	11 (5)
Clams/cockles	5 (2)
Roe	5 (2)
Abalone	3 (2)

*Fish includes multiple types of fresh fish and canned fish and different species of fish, including salmon, tuna, barramundi, kingfish, swordfish, whiting, snapper, and flathead.

### Environmental Investigation

Traceback of oysters was complex because the supply chain could include farmers, processors (harvesters), brokers, wholesalers, retailers, and food services. Brokers and processors could receive stock from multiple growers on the same day and often from different growing regions. Processors could manage multiple suppliers on the same day, and opportunities for traceability were sometimes lost because records lacked details of where batches had been distributed. Sometimes, processors recorded shuck dates but not harvest dates on packaging. There was no single method of easily identifying unlabeled oysters once original traceability was misplaced by the processor. Traceback indicated oysters had been sourced from different growing regions in South Australia, including Smoky Bay, Streaky Bay, and Coffin Bay, with the largest proportion traced back to Coffin Bay. Within Coffin Bay, there were 32 accredited growers, and it was impossible to definitively link oysters to any single grower. In total, 173 oyster exposures were able to be traced back to Coffin Bay.

Of 117 oyster samples tested for *V. parahaemolyticus,* 14 tested positive (7 from South Australia, 3 from Queensland, 3 from Victoria, 1 from Western Australia). All positive oysters were ST417. Those *V. parahaemolyticus*–positive oyster samples were from various sources, including case-patient households, retail vendors, distributors, and direct-purchase farms. We traced the original source of all 14 positive oyster samples to Coffin Bay.

The Department of Primary Industries and Regions of South Australia (PIRSA) closed the Coffin Bay growing area on November 16, 2021, for harvest of oysters. A national recall of Coffin Bay raw pacific oysters occurred on November 19, 2021, conducted via Emergency Orders under the South Australian Food Act 2001. PIRSA also served compliance orders on accredited growers in Coffin Bay on November 18, 2021, specifying legislative requirements for growers to resume harvesting. Growers were required to implement a *Vibrio* control program and provide evidence that they had infrastructure available to maintain cold chain, could address food safety requirements, could verify monitoring and traceability, and could validate refrigeration capabilities. The *Vibrio* control program required growers to place oysters under active refrigeration within 7 hours of harvest, ensure oysters were at ≤10°C within 24 hours of harvest, ensure oysters were dispatched and transported at ≤10°C, and ensure enhanced traceability by including the harvest date, area, and aquaculture license number on invoices. *Vibrio* control programs were implemented in all oyster-growing areas across South Australia during this outbreak investigation. PIRSA also conducted microbiological sampling of oysters to clear growing zones in Coffin Bay before emergency orders were able to be lifted.

### Genomic Epidemiology and Pathogenicity

Most outbreak cases (79%) could be classified as confirmed or probable cases, including 143 cases typed as ST417 and 70 cases typed as ST50. Cases of both STs occurred throughout the duration of the outbreak (Figure 1). All isolates from the 14 positive oyster samples were ST417 *V. parahaemolyticus.*

**Figure 2:**
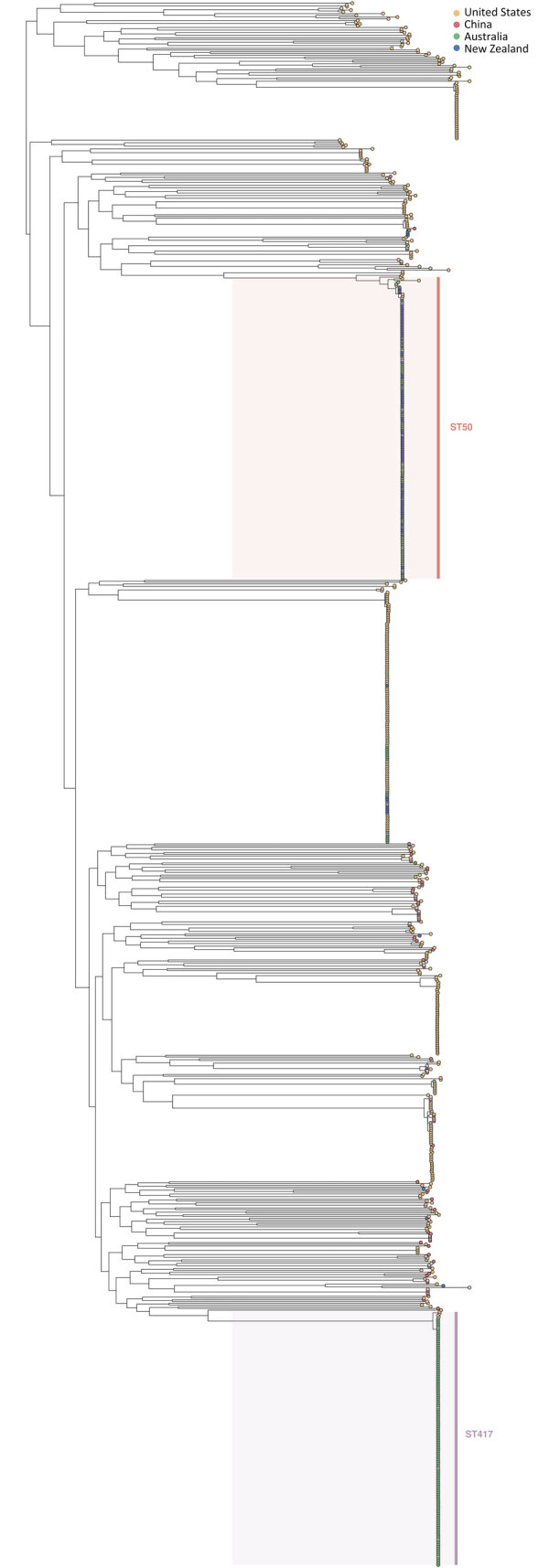
Phylogeny of *Vibrio parahaemolyticus* constructed using available pangenomes from the PubMLST database (https://pubmlst.org) and outbreak isolates of ST417 and ST 50. Data informed an investigation of *V. parahaemolyticus* foodborne illness associated with oysters, Australia, 2021–2022. ST, sequence type.

Phylogeny confirmed that ST417 and ST50 *V. parahaemolyticus* were not closely related (Figure 2). Because of the distinct nature of those strains of *V. parahaemolyticus*, we performed phylogenetic SNP clustering analyses on each ST individually. We grouped all clinical cases of ST417 and oyster samples from Australia submitted for national analysis in AusTrakka (n = 135) into a single cluster at a 5-SNP threshold. Clustering analysis of *V. parahaemolyticus* ST50 grouped 65 clinical cases at the 10-SNP threshold. Further analysis at the narrower 5-SNP threshold revealed 2 distinct but related clusters (n = 35 and n = 28; 2 were unclustered at 5 SNPs). We excluded 4 cases from the outbreak based on genomic analysis: 1 ST50 case unclustered at the 10-SNP threshold on phylogenetic analysis in AusTrakka and 3 cases that were different STs (2 ST1140 and 1 ST36).

Both STs of *V. parahaemolyticus* isolated from cases in this outbreak investigation harbored virulence genes required for pathogenicity. Specifically, all *V. parahaemolyticus* ST417 isolates harbored the virulence gene *trh*, and all ST50 harbored 2 virulence genes, *tdh* and *trh*.

## Discussion

This *V. parahaemolyticus* outbreak was caused by 2 STs and had a considerable effect on the population of Australia because of the nationwide distribution of oysters across mainland jurisdictions and cases occurring over a 5-month period. Recent investigations of other *V. parahaemolyticus* outbreaks have focused mostly on point source events, such as outbreaks on cruise ships (*8*,*20*,*21*). The communitywide outbreak in this report highlights the potential risks associated with consumption of raw oysters in Australia. Raw shellfish, particularly oysters, are known to be a common source of *Vibrio* foodborne illness, but recent trends observe increasing numbers of sporadic and outbreak cases, across an internationally wider span, somewhere cases had not been previously reported (*8*,*11*,*22*). *V. parahaemolyticus* has been isolated from other shellfish, including mussels, prawns, clams, and scallops during food surveillance studies, and has been identified as the cause of outbreaks in countries other than Australia (*3*,*23*–*25*).

The identification of 2 unrelated STs, ST417 and ST50, within this outbreak indicated the cause to be more relative to environmental factors influencing favorable growth conditions for *V. parahaemolyticus* across the oyster-growing region than to a single temperature-abuse error or single point source event. The appearance of those 2 strains could also be indicative of >1 outbreak occurring at the same time, with common contributing factors. However, multiple strains or types of a pathogen can cause discrete outbreaks and require a common public health investigation and response (*26*,*27*). The epidemiologic evidence in this investigation indicated raw oysters grown in South Australia as the cause of both ST417 and ST50 *V. parahaemolyticus* infections across Australia. Previous *V. parahaemolyticus* outbreaks have reported single-strain infections, predominantly by using traditional O and K serotyping methods (*8*,*25*). Longitudinal studies in Asia have identified a range of strains within a region (*28*), and other reports have highlighted highly virulent pandemic strains (e.g., ST36) detected across a widening international geographic range (*1*,*13*). Although minimal data are available in Australia regarding *V. parahaemolyticus* strains linked to locally acquired cases, recent increased use of genomic methods and practices will likely change that. The emergence of WGS characterization in Australia will also contribute to global knowledge regarding emerging and pathogenic strains. The presence of the *trh* virulence gene in both strains within this outbreak—and the additional *tdh* gene in the ST50 case isolates—correlates with prior literature noting that the presence of those virulence genes contributes to clinical symptoms but that both genes are not required to cause illness (*29*).

International risk assessments have been conducted for *V. parahaemolyticus* in seafood, noting the pathogenicity of the organism, the growth of *V. parahaemolyticus* increasing with increased water temperatures, and the need for strict postharvest controls to reduce the risk for foodborne disease (*6*,*12*). Outbreaks have occurred more frequently during warmer months (*3*) and at times when seawater temperatures have increased (*8*,*20*) or other environmental factors have had an influence (e.g., El Niño events or decreases in salinity) (*10*,*30*). In response to the outbreak we have described, oyster growers implemented postharvest controls through a *Vibrio* control program, where oysters were placed under active refrigeration. General trends of increased sea surface temperatures in Australia (*31*) and the seasonal occurrence of the Leeuwin current, which brings warm tropical waters to Western and South Australia (*31*), potentially created favorable conditions for growth of *V. parahaemolyticus* in South Australia oyster-growing bays. We believe that further research would improve understanding of risk factors for *V. parahaemolyticus* outbreaks in Australia’s prone regions, including ongoing environmental surveillance at harvest sites to monitor seawater temperatures, salinity, and harvest conditions, as well as at points along the storage and transport chain to consumers.

We noted that many outbreak case-patients in this study consumed medication that reduces stomach acid, a finding noted in previous studies that investigated risk for *V. parahaemolyticus* and other bacterial gastroenteric infections (*32*,*33*). Gastric acidity also decreases with age (*34*); therefore, infection susceptibility could increase with age, which is consistent with our observed median case patient age of 52 years. The fact that a large portion of our case-patients were older adults might also be related to food consumption patterns in the general population of Australia, where mollusks are less commonly eaten by children compared with adults (*35*). The outbreak we studied showed higher severity of illness than some previous outbreaks; for example, we noted 13% of case patients hospitalized and a single case with septicemia, compared with a study that investigated an outbreak associated with Alaska oysters, where there were no hospitalizations (*8*). Conversely, we noted a lower hospitalization rate for case-patients (13%) compared with a longer-term study that reported a hospitalization rate of 44% (*9*). Individual factors and the pathogenicity of different strains could affect disease severity in outbreaks.

The first limitation of our investigation is that culture for *V. parahaemolyticus* is not always attempted on diarrheal samples in diagnostic laboratories in Australia, and *V. parahaemolyticus* targets are often omitted in routine fecal multiplex PCR kits employed for direct detection of enteropathogens. Also, there was likely underreporting of cases because *V. parahaemolyticus* is not a notifiable condition in all jurisdictions in Australia. However, public health laboratories were contacted by their respective health departments and asked to provide information on enteric *V. parahaemolyticus* cases. Further typing of strains by WGS is also not consistently conducted across Australia and sometimes must be specifically requested if an outbreak is suspected. During this outbreak, some requests for further typing were made several weeks after the initial isolation, at which point no specimens were available for shipment to the public health laboratories. Because of incomplete typing of all case isolates in this outbreak, some cases might have been of a different ST and might not have been specifically linked to the current outbreak. In addition, *V. parahaemolyticus* outbreak cases we studied coincided with a national surge in SARS-CoV-2 infections in Australia, putting strain on public health resources. Therefore, not all case-patients were able to be interviewed, and not all isolates were able to be further typed.

There were also limitations in the traceback of oysters within a complex supply chain, including distributors and retailers receiving stock from multiple growers and different growing regions, leading to potential mixing of stock, some incomplete records and invoicing, and case-patients having multiple exposures to oysters within their incubation period. Mixing of oyster stock could also have contributed to the identification of multiple strains of *V. parahaemolyticus* in cases included in this outbreak. Although traceback was unable to be completed for all cases, oysters consumed by most casepatients were traced to at least the harvest area. 

In conclusion, evidence for the source of this outbreak was strong, considering the oyster consumption among case-patients, traceback of the source of oysters consumed by case-patients, and identification of the same strain of *V. parahaemolyticus* in both oysters and case patients. The reduction in cases of *V. parahaemolyticus* after the recall of oysters and wide implementation of *Vibrio* control programs supports this evidence. This outbreak of *V. parahaemolyticus* associated with consumption of Australia-grown oysters, largely consumed raw, has led to improvements in postproduction control and traceability in the oyster industry in South Australia. The outbreak also spotlighted the virulent potential of *V. parahaemolyticus* and the value in distinguishing it as a nationally notifiable disease in Australia. Improved surveillance data, including strain identification from a wider range of regions, and a clearer understanding of underreporting have been highlighted by the World Health Organization as priorities for improving risk assessment processes for *V. parahaemolyticus* (*22*). Increased surveillance across all jurisdictions in Australia would improve outbreak detection and ensure a prompt and coordinated public health response.
